# Supplementary material for: Distinct homotypic B-cell receptor interactions shape the outcome of chronic lymphocytic leukaemia
Source: Nat Commun. 2017 Jun 9;8:15746. doi: 10.1038/ncomms15746 (PMC5472768; doi:10.1038/ncomms15746)
Supplement: Supplementary Information — Supplementary figures and supplementary tables. [file ncomms15746-s1.pdf]

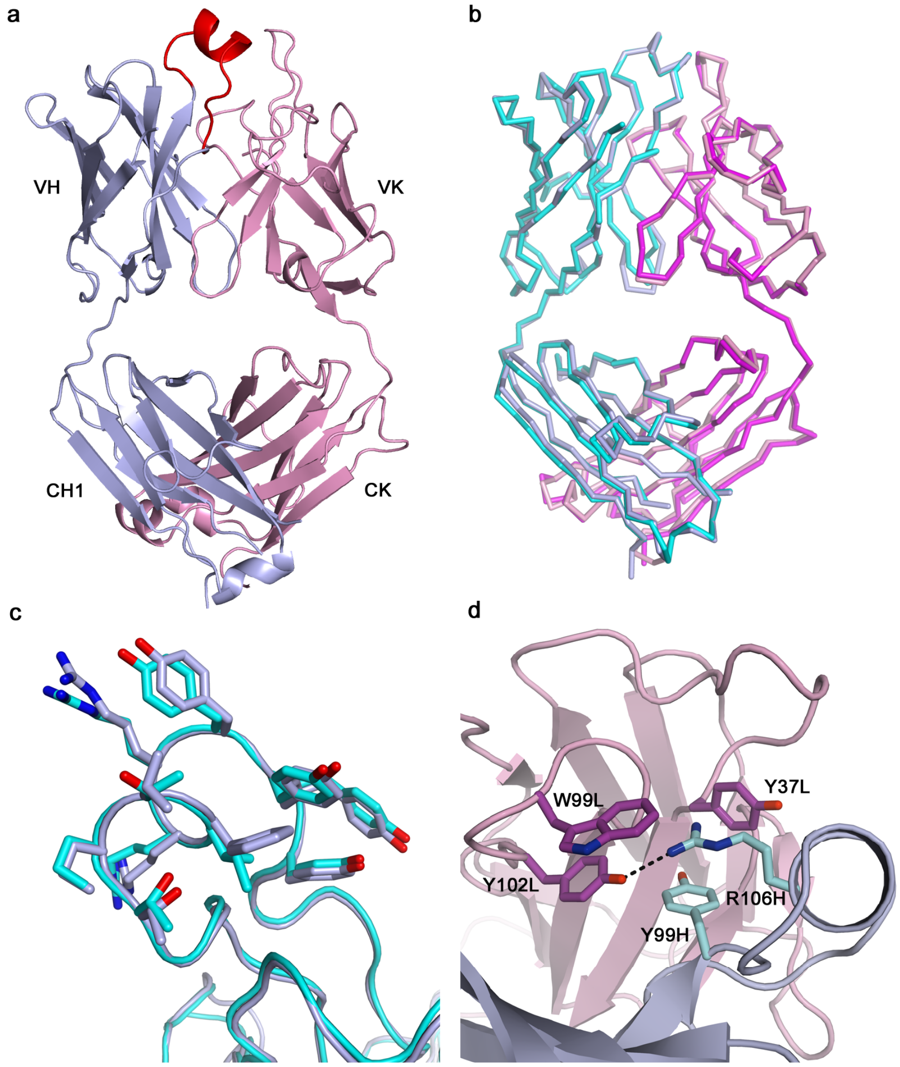

Supplementary Figure 1 | Structural Properties of Subset 4 BcR Fabs. **(a)** Overall structure of the subset #4 CLL IgG Fab fragments. The heavy and light chains are colored light blue and pink, respectively. The HCDR3 loop of the receptor (red) is folded into a characteristic helical conformation. **(b)** Superposition of the subset #4 BcR IG Fab structures. CLL240 (heavy chain colored light blue, light chain pink) and CLL183 (heavy chain colored cyan, light chain magenta) have a r.m.s.d. of 0.60 Å for 415 Cα atoms. **(c)** Detail of the HCDR3 conformation for CLL240 (carbon atoms light blue) and CLL183 (carbon atoms cyan). Differences in the position of the atoms are within coordinate errors. **(d)** Subset #4 BcR IGs include a conserved RRYYYY motif in the HCDR3 loop. The first Arg residue protrudes into a hydrophobic cage lined by residues from both the heavy and light chain. Only the *IGKV2-30*/*IGKJ2*01* rearrangement codes for a protein with all aromatic residues, thus explaining the light chain bias in the subset #4 receptors.

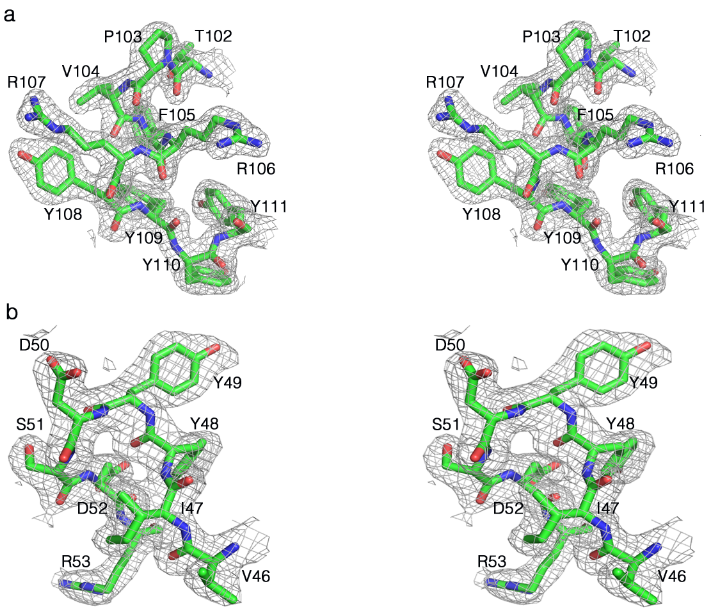

Supplementary Figure 2 | Sample electron density for the CLL240 and P11475 Fabs. (a) Stereo view of the electron density map for the subset #4 CLL240 BcR Fab. The HCDR3 loop (residues 104-111) is shown as a stick model. **(b)** Electron density for the LCDR2 loop in the Fab subset #2 P11475. In both panels, the electron density was calculated using σ_A-weighted 2mF_o-DF_c coefficients and model phases, contoured at 1.2σ and displayed with a carving radius of 2.0 Å.

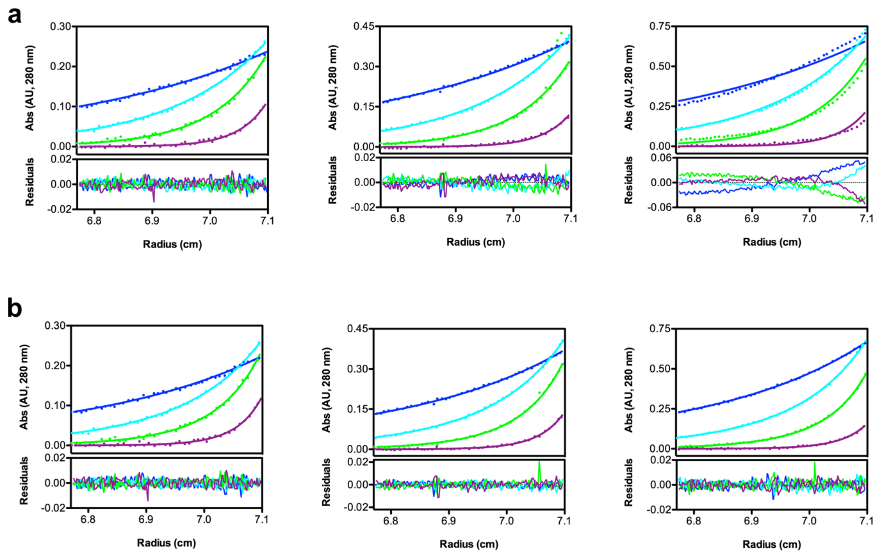

Supplementary Figure 3 | Analytical Ultracentrifugation Data Analysis for CLL-derived Fabs. Sedimentation equilibrium radial absorption curves collected as described in the Experimental Procedures section, and fitted with mathematical models assuming a monomeric Fab in solution **(a)** or self-association **(b)**. For clarity, one point every four measured is shown. Each panel corresponds to a CLL183 Fab concentration (left to right 0.1, 0.15, and 0.26 $mg \cdot ml^{-1}$, corresponding to 2, 3, and 5.2 µM). The curves represent the fitting for each velocity (5161 blue, 11612 cyan, 20644 green, and 39030 g violet). The residuals are low and randomly distributed around zero for the self-association model, while the fitting for the monomer is suboptimal and shows systematic trends with increasing protein concentration, a behaviour consistent with the formation of oligomers. See also Supplementary Table 2 for fitting comparisons. Similar results have been obtained for the CLL240 Fab.

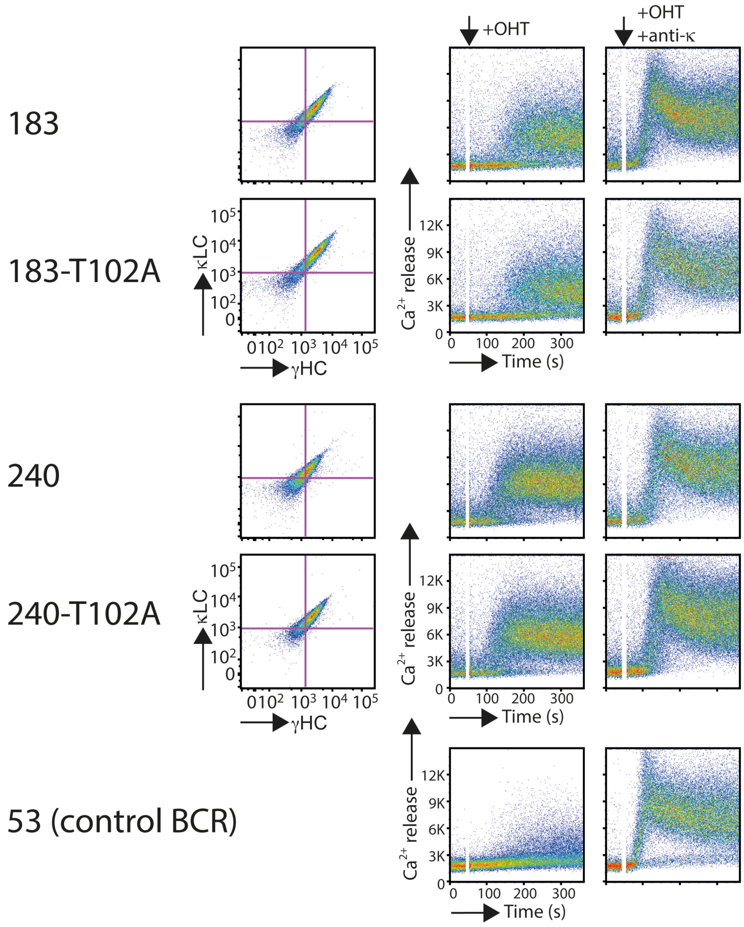

Supplementary Figure 4 | Specificity of the HCDR3-mediated homotypic interaction in subset 4 receptors. Mutation of a HCDR3 residue not involved in homotypic interaction in subset #4 BcRs CLL183 and CLL240 does not affect the Ca^{2+} mobilization upon 4-OHT stimulation. The amino acid T^{102H} is exposed to the solvent, but not directly involved in stabilizing intermolecular interaction. Its mutation to alanine does not affect the Ca^{2+} influx.

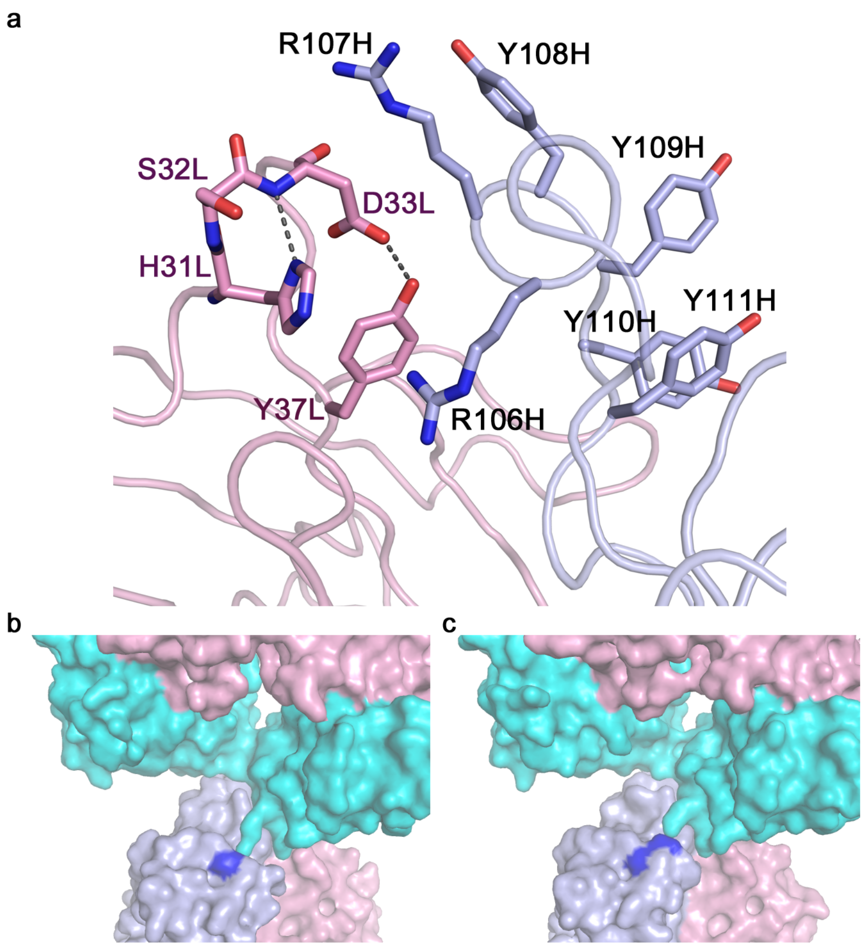

Supplementary Figure 5 | Self Recognition of Subset #4 CLL BcR IGs is Affected By SHMs. The CLL-biased somatic hypermutations (SHMs) in subset #4 Fabs affect the receptor structure and stability. **(a)** In both CLL183 and CLL240, the SHM-introduced His^{31L} is hydrogen bonded to the VK CDR1 loop. Consequently, residue Asp^{33L} is poised to orient the side chain of Tyr^{37L}, part of the aromatic cage that accommodates Arg^{106H} and stabilizes the VH CDR3, facilitating self-recognition. **(b) and (c)** Comparison of the interaction surfaces of CLL183 (**b**) and CLL240 (**c**) Fabs. The CLL240-derived BcR bears residue Glu^{31H} deriving from subset-biased SHM, while in CLL183 this residue is the germline-encoded Gly. The side chain of the Glu residue approaches the opposite BcR molecule, and contributes to a larger interaction surface (597 vs. 558 Å^2) with the epitope.

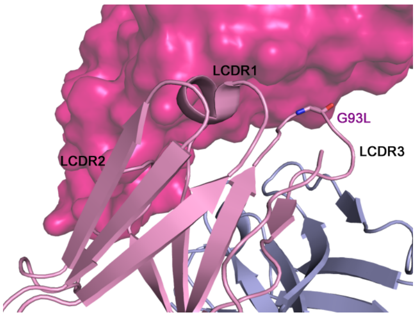

Supplementary Figure 6 | A Recurrent SHMs in Subset 2 BcRs Allows Intimate Contacts Between the Receptors. In the *IGHV3-21*/*IGLV3-21*-expressing subset #2 BcRs, recurrent subset-specific SHMs have been observed. The recurring Ser^{93L}Gly mutation is located at the tip of LCDR3, and it is present in the crystallized Fab fragment of the P11475 receptor. The Cα atom of the Gly residue is in van der Waals' contact with residues Pro^{7L} and Val^{10L} of the opposing receptor (3.9 and 4.5 Å distances, respectively). The germline-encoded Ser would at least induce a local structural rearrangement to retain a similar mode of homotypic interaction. Thus, the introduction of a Gly from the germline sequence at this position likely facilitates the observed BcR-BcR interactions.

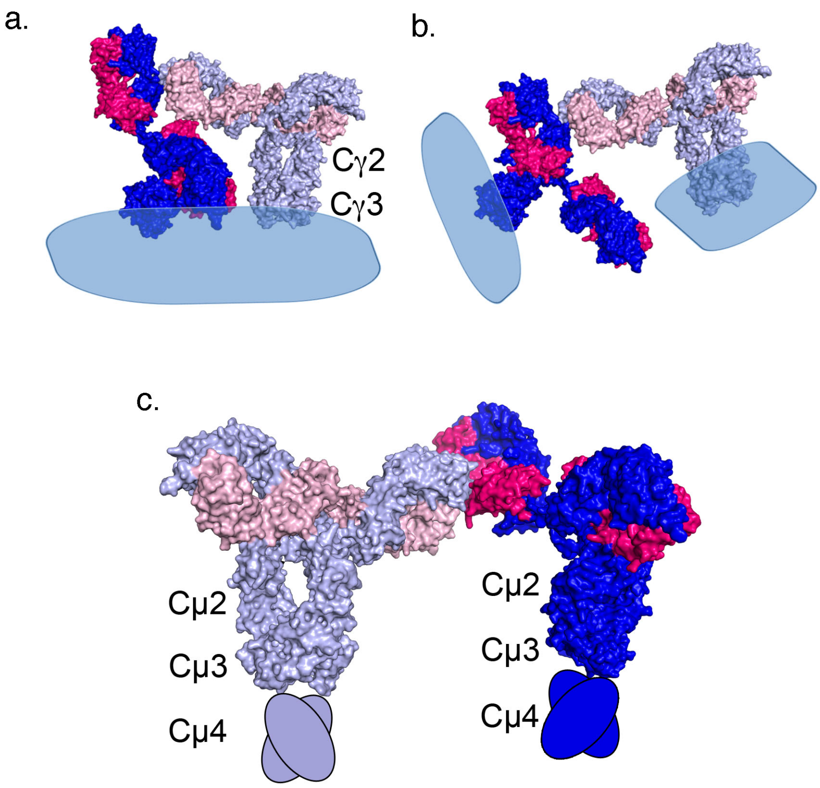

Supplementary Figure 7 | Molecular model for homotypic interactions between BcRs derived from subset 4 and subset 2 CLL cases. Models for the complete BcRs were generated using the crystal structure of the full-length B12 IgG (PDB code 1HZH). The variable domains of the complete IgG structure were superimposed with the corresponding domains of the CLL-derived BcR Fab fragments crystallized using PYMOL. In all figures, the "antigen" molecule is shown with the molecular surface representation, colored blue in the heavy chain and magenta in the light chain. The surface is instead colored light blue and pink for the BcR providing the paratope in the interaction. The subset #4-derived BcRs can interact both in cis **(a)** and in trans **(b)**. In these panels, planes roughly perpendicular to the twofold axis relating the Cγ2 and Cγ3 domains are shown to facilitate the viewing of the BcR orientations. **(c)** A model for the intermolecular interaction in the subset #2 IgM shows that the receptors may interact while expressed on the same cell. It should be noted that this model is for visualization purposes only, as its validity is limited due the lack of complete IgM structures. It should be noted that the orientation of the Fab fragments with respect to the Fc portion can be readily varied through rearrangements of the peptide linking the C_{H1} and C_{H2} domains, thus other geometries of interaction are in principle possible.

a

b

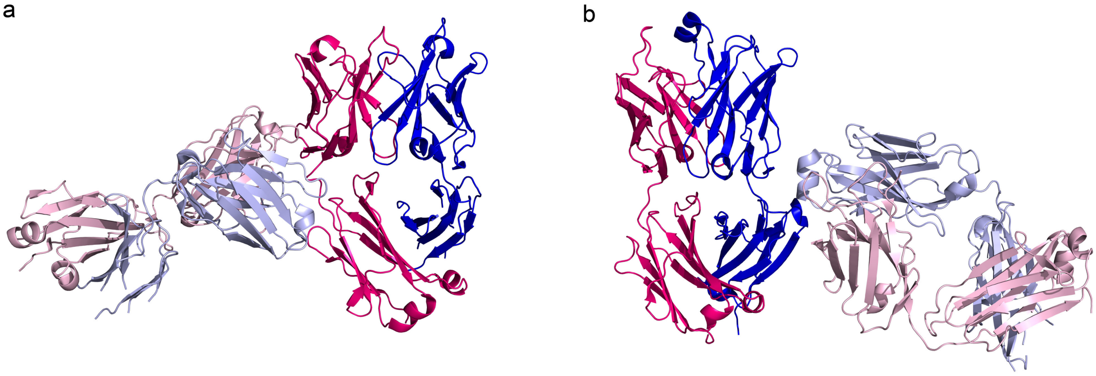

Supplementary Figure 8 | Side-by-side comparison of the homotypic interactions in subset 2 and subset 4 stereotyped CLL cases. (a) Ribbon representation of the P11475 Fab fragments, showing the interaction between the two molecules. The light chain (colored pink) CDR2 loop contacts the region linking the VL-CL linker peptide of the opposing molecule (in magenta). **(b)** Ribbon representation of the CLL240 Fab fragments, showing that the helical HCDR3 loop of the heavy chain (colored light blue) interacts with a composite epitope spanning the VH and Cγ1 domains (colored blue). In both panels, the "antigen" molecule is presented with a vertical orientation of the pseudo-twofold axis relating the VH and VL domains. The interaction epitopes are spatially and chemically distinct.

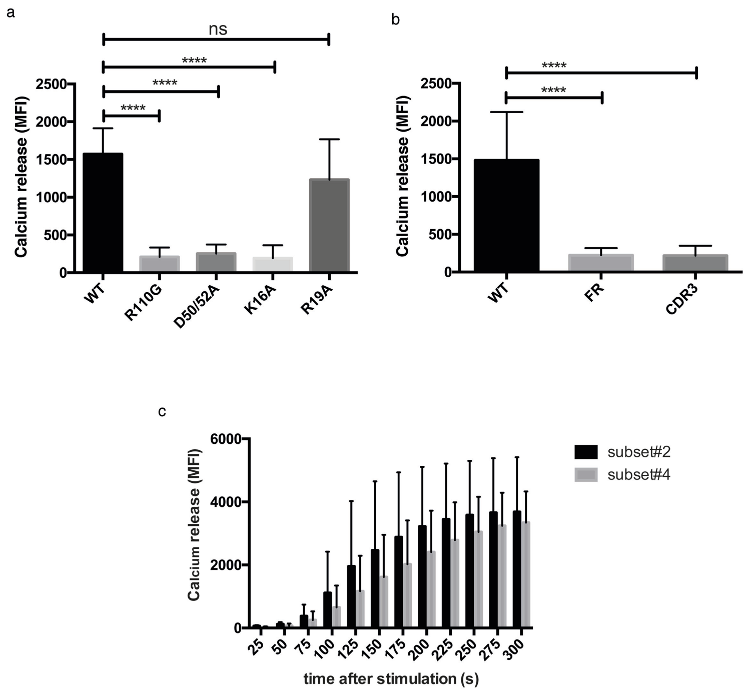

Supplementary Figure 9 | Quantitative analysis of the Ca^{2+} influx in CLL BcR-transfected TKO cells. The mean fluorescence intensity of tamoxifen-stimulated TKO cells transfected with BcRs derived from either subset #2 or subset #4 cases was analyzed. **(a)** The mutations engineered to disrupt the interactions observed in the crystal structure of the P11475 BcR significantly reduce the total Ca^{2+} influx in TKO cells, while the control mutation R19A ($Arg^{19L}Ala$) does not affect the ion flux. **(b)** Similarly, the mutations that affect the paratope-epitope interactions in the subset #4-derived BcRs severely reduce the Ca^{2+} mobilization. **(c)** Kinetic analysis of the Ca^{2+} influx in TKO cells transfected with subset #2 (black bars) or subset #4 (grey bars) receptors. Overall, the less stable homotypic interactions between subset #2 BcRs display a trend for larger mobilization of Ca^{2+} compared to the stable intermolecular interactions in subset #4. Despite this clear trend, statistical significance is not reached. Nevertheless, Ca^{2+} influx is an early event in antigen-mediated B-cell signaling upon PLCγ activation, and induction of anergy in primary, patient-derived cells may be a consequence of this persistent stimulation over long periods of time. Values are means from three separate cultures, and the error bars shown are standard deviations.

Supplementary Table 1. Buried surface area for Fab-antigen complexes.

Antibody	Antigen	PDB ID	BSA (Å^2)
NC10	Neuraminidase	1A14	712
D.13	Lysozyme C	1A2Y	704
Fab59.1	gp120	1ACT	563
Bevacizumab	VEGF	1BJ1	859
CabRn05(Fv)	RNAse	1BZQ	555
13B5	p24 capsid protein	1E6J	662
Bion-1	Common β chain	1EGJ	767
Ru5	Von Willebrand factor	1FE8	987
26-2F	Angiogenin	1H0D	715
Rheumatoid factor	IgM	1ADQ	749
Average value ± s.d.			727±128
CLL183		5DRW	558
CLL240		5DRX	597
P11475		5IFH	556

Buried surface areas for ten selected antibody-protein antigen structures present in the Protein Data Bank (http://www.rcsb.org/pdb). Surface values were calculated using the PISA server (http://www.ebi.ac.uk/pdbe/pisa). BSA, buried surface area. For reference, the values for the CLL-derived BcR Fab fragments in the present study are also shown (not included in the calculation of the average value and standard deviation).

Supplementary Table 2. Fitting of CLL subset #4 Fabs SE-AUC data with different models.

BcR Fab	Data fit model	Residual chi^2	K_D ± s.d. (µM)
CLL183	Monomer	2.472	-
	Self-association	0.974	15.6±0.8
CLL240	Monomer	7.495	-
	Self-association	1.557	14.0±0.4

Affinity and kinetic stability of self-association for CLL-derived BcR IGs. For the subset #4 BcRs the results of the fitting of SE data from different models are shown for comparison. The analysis was performed with the program SEDPHAT.
